# Transcriptome Responses to Defined Insecticide Selection Pressures in the German Cockroach (*Blattella germanica* L.)

**DOI:** 10.3389/fphys.2021.816675

**Published:** 2022-02-04

**Authors:** Michael E. Scharf, Zachery M. Wolfe, Kapil R. Raje, Mahsa Fardisi, Jyothi Thimmapuram, Ketaki Bhide, Ameya D. Gondhalekar

**Affiliations:** ^1^Department of Entomology, Purdue University, West Lafayette, IN, United States; ^2^Bioinformatics Core, Purdue University, West Lafayette, IN, United States

**Keywords:** cockroach genome, gregarine, baculovirus, resistance, P450, FE4 esterase

## Abstract

Cockroaches are important global urban pests from aesthetic and health perspectives. Insecticides represent the most cost-effective way to control cockroaches and limit their impacts on human health. However, cockroaches readily develop insecticide resistance, which can quickly limit efficacy of even the newest and most effective insecticide products. The goal of this research was to understand whole-body physiological responses in German cockroaches, at the metatranscriptome level, to defined insecticide selection pressures. We used the insecticide indoxacarb as the selecting insecticide, which is an important bait active ingredient for cockroach control. Six generations of selection with indoxacarb bait produced a strain with substantial (>20×) resistance relative to inbred control lines originating from the same parental stock. Metatranscriptome sequencing revealed 1,123 significantly differentially expressed (DE) genes in ≥two of three statistical models (81 upregulated and 1,042 downregulated; FDR *P* < 0.001; log2FC of ±1). Upregulated DE genes represented many detoxification enzyme families including cytochrome-P450 oxidative enzymes, hydrolases and glutathione-*S*-transferases. Interestingly, the majority of downregulated DE genes were from microbial and viral origins, indicating that selection for resistance is also associated with elimination of commensal, pathogenic and/or parasitic microbes. These microbial impacts could result from: (i) direct effects of indoxacarb, (ii) indirect effects of antimicrobial preservatives included in the selecting bait matrix, or (iii) selection for general stress response mechanisms that confer both xenobiotic resistance and immunity. These results provide novel physiological insights into insecticide resistance evolution and mechanisms, as well as novel insights into parallel fitness benefits associated with selection for insecticide resistance.

## Introduction

The German cockroach, *Blattella germanica* L. is an international urban pest that affects millions of residences on a global scale (Vargo, 2021). *B. germanica* impacts human health through the production of asthma and rhinitis-causing allergens, transmission of food-borne pathogens and psychological stress (Kopanic et al., 1994; Elgderi et al., 2006; Sohn and Kim, 2012). Up to 85% of inner city homes in the United States test positive for cockroach allergens and 60–93% of inner-city children with asthma are sensitized to cockroaches (Gore and Schal, 2007; Do et al., 2016; Pomés et al., 2017). Cockroaches also host pro- and eukaryotic microbes that contribute to house-dust microbiomes that intensify asthma (Roth and Willis, 1960; Pai et al., 2003; Carrasco et al., 2014; Crawford et al., 2015; Thorne et al., 2015; Wada-Katsumata et al., 2015; Lai, 2017; Turturice et al., 2017).

Insecticides are essential for efficiently overcoming health impacts of cockroaches (Schal and Hamilton, 1990; Pomés et al., 2017). However, insecticide resistance has been a formidable recurring barrier to effective cockroach control for decades (Scharf and Gondhalekar, 2021). As of 2016, the German cockroach was reported as having developed resistance worldwide to 42 distinct insecticide active ingredients in at least 219 documented cases (Zhu et al., 2016). Because cockroaches live in relatively closed populations (Crissman et al., 2010; Vargo et al., 2014; Vargo, 2021), resistance can build quickly, even with moderate insecticide selection pressure (Scharf et al., 1997a,b; Gondhalekar et al., 2013; Fardisi et al., 2019). Cockroach baits are widely used in management programs and have been an effective tool for controlling cockroaches and reducing pesticide loads in urban housing (e.g., Miller and Smith, 2020); however, resistance can readily develop even to bait insecticides (Gondhalekar et al., 2011, 2013, 2016; Gondhalekar and Scharf, 2012, 2013; Ko et al., 2016; Fardisi et al., 2019).

The goal of this research was to use a quantitative metatranscriptomics approach to better understand whole-body physiological responses in *B. germanica* to defined insecticide selection pressures. The insecticide indoxacarb was used as the selecting insecticide, which is an important bait active ingredient for cockroach control (Appel, 2003; Buczkowski et al., 2008; Gondhalekar et al., 2011). Through previous studies we documented early stages of resistance evolution to indoxacarb among field populations (Gondhalekar et al., 2011, 2013) and verified hydrolysis and cytochrome P450-based oxidation as important steps in indoxacarb bioactivation and detoxification (Gondhalekar et al., 2016). Our specific objectives here were to: (1) identify whole-body mRNA expression profiles and candidate genes associated with indoxacarb resistance, and (2) investigate a subset of candidate resistance-associated genes in an independent and highly resistant field strain. Our findings reveal novel physiological insights into insecticide resistance evolution in this important global health pest; mainly that resistance evolves rapidly as a complex phenotype with multiple underlying mechanisms that include both xenobiotic detoxification and microbial clearance.

## Materials and Methods

### Cockroach Strains and Rearing

**FIGURE 1:**
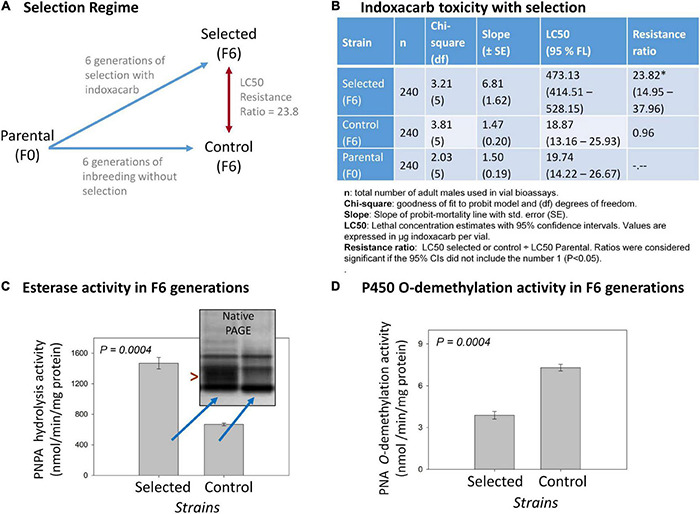
Overview of the indoxacarb selection process and key associated findings. **(A)** Diagram of the selection regime over six generations resulting in a F6-selected strain having 23.3-fold indoxacarb resistance. **(B)** Concentration-response vial bioassay results on selected F6, control F6 and Parental F0 lines showing LC50 determination outcomes and resistance ratios. **(C)** Cytosolic esterase activity in the F6 selected and F6 control lines using the model substrate *p*-Nitrophenylacetate (PNPA) and also by native PAGE visualization (inset). **(D)** Microsomal cytochrome P450 *O*-demethylation activity in the F6 selected and F6 control lines using the model substrate *p*-Nitroanisole (PNA).

The Arbor Park (AP) strain was used for indoxacarb selection experiments within 12 months of its collection and is the source of the “Parental-F0,” “Selected-F6,” and “Control-F6” lines (Figure 1A). The AP strain was collected from an apartment in Gainesville, FL, United States after control failures with multiple insecticide products. The highly resistant field-collected “Oviedo-R” strain and the standard susceptible Johnson Wax (JWax-S) strain were included in *post hoc* validation experiments. The Oviedo-R strain was collected from a restaurant near Orlando, FL, United States, where indoxacarb-containing cockroach baits were used regularly for at least 3–4 years and was tested within 6 months of its collection. The JWax-S strain has been in culture for over 70 years and has never been exposed to synthetic organic insecticides. All of the above cockroach strains were reared in mixed life stages in 1,000 cm × 400 cm × 300 cm (L × W × H) plastic boxes with aerated lids, greased walls, cardboard harborage, and an *ad libitum* water source and rodent diet (#8604, Harlan Teklad, Madison, WI, United States). Rearing conditions consisted of a 12:12 (L:D) photocycle, 25–27°C and ∼50% RH.

### Chemicals

Technical grade indoxacarb (99.1% AI), blank bait matrix and formulated gel bait product containing indoxacarb (Advion®) were provided by DuPont Inc. (Wilmington, DE, United States). All solvents and buffer components were purchased from Fisher Scientific (Pittsburgh, PA, United States) or Sigma (St. Louis, MO, United States). Other chemicals used in enzyme assays and other procedures are detailed in a previous report (Gondhalekar, 2011). These chemicals include the P450 substrate *p*-Nitroanisole, the esterase substrates *p*-nitrophenyl acetate and naphthyl acetate, the GST substrate chloro-dinitro benzene, protein extraction buffers, and native PAGE reagents and stains.

### Selection Procedures

For selection experiments, >1,200 large nymphs (4th to 5th instar) were separated from the AP lab cultures and divided into six sub populations of ca. 200 nymphs. These life stages were used because they are among the most tolerant cockroach life stages (Koehler et al., 1993). A feeding delivery method was used for selections because it exactly represents field exposure to indoxacarb baits (Gondhalekar et al., 2011, 2013; Gondhalekar and Scharf, 2012). In brief, pellets of blank gel bait matrix (ca. 5–10 mg wet weight) were prepared manually and treated with a dose of indoxacarb in acetone that provided ca. 60 to 80% mortality (Supplementary Table 1). Large nymphs that were pre-starved for 24-h were held individually with a single indoxacarb treated pellet in 1 oz. (30 mL) cups with vented lids. After 3 days, nymphs that had completely eaten bait pellets were transferred in groups of 100 into 17.8 cm × 17.8 cm × 6 cm disposable plastic Glad^®^ boxes under conditions detailed above, where they remained for 7–10 days. Almost all nymphs exhibited intoxication symptoms at 3 days; however, ca. 25–35% completely recovered over the next 7–10 days. These surviving individuals were reared to adulthood and used as founders for the next generation (Scharf et al., 1997b). Selections continued in this manner for six generations (Parental or F0 to F5 generation). The above process happened with three replicate “selected” and “control” lines; control lines received lab diet only. Details of indoxacarb doses used and percent survival for each round of selection are given in Supplementary Table 1.

### Bioassays

Bioassays performed included vial and feeding bioassays, each in two formats.

#### Vial Bioassays

Vial bioassays were done in concentration-response and diagnostic-concentration formats. Concentration-response bioassays were done with adult males from the Parental (F0), Control F6 and Selected F6 generations following established protocols (Gondhalekar et al., 2011; Fardisi et al., 2019). Four to five concentrations providing 10–90% mortality were used for calculating lethal concentration (LC) estimates and associated parameters using probit analysis (see below). Diagnostic concentration bioassays were done by testing adult males of the JWax-S and Oviedo-R strains at previously-established concentrations of 30 and 60 μg indoxacarb per vial (Gondhalekar et al., 2011, 2013). Three replicates of ten insects were conducted per concentration and isolate (*n* = 90). Control vials received acetone only.

#### Feeding Bioassays

Feeding bioassays were done in dose-response and no-choice formats. Dose-response bioassays were conducted with Parental (F0), Control F6 and Selected F6 generation adult males using a published protocol (Gondhalekar et al., 2011). At least four doses producing 10–90% mortality were tested against each sub-population. Each replicate had ten insects and bioassays were repeated three times for each dose and sub-population. The dose-response data were analyzed by probit analysis (details below) and used for calculating lethal doses (LD) and associated parameters. Realized heritability (*h*^2^) estimation was done on oral dose-response data to determine the proportion of phenotypic variance in resistance caused by additive genetic variation (Falconer, 1989; Tabashnik, 1992).

No-choice assays used formulated indoxacarb gel bait product (0.6% indoxacarb) with adult males of JWax-S strain and the field-selected highly resistant Mid-Florida strain using published protocols (Gondhalekar et al., 2011). The same protocols were followed for control treatments that were conducted using blank gel bait without indoxacarb. These tests were done on lab-reared individuals within 6 months of collecting the Oviedo-R strain. In these tests no alternative/competing food other than gel bait was present in the bioassay arenas. No-choice bait feeding assays were preferred for testing the Oviedo-R strain because within 6 months after field collection the population numbers were relatively low and the bait feeding bioassay required less insects as compared to the traditional dose or concentration-response tests. Disposable plastic GladWare boxes (17.8 cm × 17.8 cm × 6 cm; Clorox Co., Oakland, CA, United States) served as bioassay arenas and were provisioned with a water source, harborage and 0.5 g of formulated indoxacarb gel bait product in a plastic dish (Gondhalekar et al., 2011). Additional bait or blank matrix was provided if the insects consumed a majority of the initially provided bait. Mortality was recorded at 1, 3, 7, 14, and 21 days. Five replicates with 10 adult males per replicate (*n* = 50) were performed for each strain.

### Enzyme Activity Assays

Enzyme assay methods were detailed previously (Gondhalekar, 2011). Esterase (hydrolysis) and P450 (*O*-demethylation) activity assays were done on F6-selected and control lines using the model substrates *p*-nitrophenyl acetate and *p*-nitroanisole, respectively. Esterase native PAGE was done using the model substrate beta-naphthyl acetate. Esterase and P450 investigations were done on soluble and microsomal protein preparations, respectively, made from the same insect homogenates. Protein content was normalized using a commercial Bradford assay (Bio-Rad) with bovine serum albumin as a standard. All enzyme and protein assays were performed in triplicates representing three F6-selected and three control lines.

### Transcriptome Sequencing

Total RNA was isolated from three independent biological replicate samples of 10 whole adult male cockroaches from each of F6-selected and control lines. A two-step process was used that included the Promega SV Total RNA Isolation Kit (Madison, WI, United States) followed by the Bioline TRIsure kit (Taunton, MA, United States). Manufacturer protocols were followed for both kits with the exception that DNase treatment was excluded as the final step for the second kit. The use of two kits ensured the RNA samples were free of excess protein. Total RNA yields ranged from 5.8–18.6 μg with A260/280 and 260/230 ratios in the range of 1.8–2.1. Sample quality was further assessed using an Agilent Bioanalyzer (Agilent Technologies, Santa Clara, CA, United States) which verified above yields and provided acceptable RIN scores of 7.20–7.76. RNA samples were enhanced for messenger RNA (mRNA) using the Agilent Tru-Seq RNA prep kit before bar-coded sequencing libraries were made. Sequencing was done on the Illumina HiSeq 2000 platform by the Purdue University Genomics Core (West Lafayette, IN, United States). Sequencing reads were filtered using Phred quality scores and other parameters, and *de novo* transcriptome assembly performed from all six pooled replicate samples (3 selected and 3 control) using Trinity (Grabherr et al., 2011). Paired reads for individual replicate samples were then mapped to the *de novo* transcriptome using Bowtie2 (Langmead and Salzberg, 2012), which provided read counts used for differential expression analyses as detailed below. The *de novo* transcriptome assembly was used because a reference German cockroach genome was not yet available at the time sequencing was completed; however, new blast searches with significant contigs were performed in 2021 which confirmed origins in either the *B. germanica* genome or from other microbial sources.

### GO and KAAS Annotation Analyses

The assembled contiguous sequences, i.e., “contigs” were analyzed by BLAST, Blast2GO and KAAS to assign identities and functional annotations. The contigs, as well as single-read “singletons,” were annotated using “Blast2GO” for cellular location (CL), biological process (BP), and molecular function (MF) (Conesa et al., 2005). BLAST searches were performed against the Genbank “nr” database available as of May 2012 at www.ncbi.nlm.nih.gov and re-verified in June 2021 (file provided). KAAS analysis was done to gain insights into possible pathways and gene networks involved in resistance (Moriya et al., 2007). KAAS is a rapid method that establishes orthologies to genes operating within conserved pathways using best hit information and Smith–Waterman scores.

### Differential Expression Analysis Methods

Basic exploration of the data such as accessing data range, library sizes etc. was performed to ensure data quality. Three models were used for analysis of read-count data obtained for each contig and singleton: edgeR (v 2.9), DESeq (v 1.8.3), and voom-limma (v 1.2.0). An edgeR object was created by combining the counts matrix, library sizes, and experimental design (3 replicates each for selected and control lines, i.e., “samples”) using the edgeR package. Normalization factors were calculated for the counts matrix, followed by estimation of common dispersion of counts. An Exact test for differences between the negative binomial distribution of counts for the selected and control replicates resulted in finding differential expression, which was then adjusted for multiple-hypothesis testing to generate a result file. A DESeq object analogous to the aforementioned edgeR object was created and used to generate normalization factors followed by dispersion estimates using DESeq package. The DESeq method tests for differences between the base means of the experimental conditions and differential expression (DE) results were reported in another result file (DE_analysis_DESeq.csv). A third method called ‘voom’ from the limma package was also used for DE analysis. The ‘voom’ function carries out log2 transformation of counts followed by mean-variance estimation and assigns weight to each transformed value. Linear model coefficients were then calculated using limma’s design matrix and log2 transformed values. The linear model was fitted using an empirical Bayes method and differences between counts between selected and control replicates were calculated, which was then adjusted for multiple-hypothesis testing, and reported as result file. Venn diagrams were generated displaying the DE contigs with false discovery rate (FDR) *P* < 0.05 that were found to be common among all three analysis methods using the online tool Venny^1^.

### Quantitative Real-Time PCR

Expression levels of 48 significantly up- and down-regulated genes identified from Illumina sequencing (Table 1) were investigated for validative purposes by qRT-PCR in the F6-selected and control lines (three biological replicates each). PCR primers for target and reference genes were designed using Primer 3^2^ (Untergasser et al., 2012) and are given in Supplementary Table 2. The efficiencies of qPCR primers used in this experiment were empirically determined and they were within the recommended range of 90–110%. Validative qRT-PCR was done on aliquots of the same RNA preparations used for Illumina sequencing above using an iCycler iQ real-time PCR detection system (Bio-Rad, Hercules, CA, United States) with Sybr Green product tagging (2x SensiMix Sybr and Fluorescein Kit; Quantace, Norwood, MA, United States). Each 20 μL qRT-PCR reaction in a 96-well format consisted of 10 μL SensiMix (Bioline, Taunton, MA, United States), 7 μL nanopure water, 1 μL each of forward and reverse primer (0.5 μM final concentration) and 1 μL cDNA. A published qRT-PCR temperature program was followed (Scharf et al., 2008). Three technical replicates were performed for each gene and cDNA preparation. The resulting critical threshold (*C*q) data were analyzed by the 2^–ΔCTΔCT^ method (Livak and Schmittgen, 2001).

**TABLE 1:** An overview of 53 transcript contigs that were significantly differentially expressed with selection for indoxacarb resistance.

Contig no.	Best blastX match (Genbank, 2021)^1^	Best *Blattella germanica* genome match	Fold change	FDR adj. *P*-Value	Contig length
1*	Hypothetical protein C0J52_04259 (*Blattella germanica*) short match of 92%	Hypothetical protein C0J52_04259 (*B. germanica*) short 92% match	83.94	0.00009	792
2*	PiggyBac transposable element-derived protein 3 (*Cryptotermessecundus* LOC111875436)	No match	67.05	0.00001	501
3*	Cytochrome P450 6k1 (*Blattella germanica*)	97% match to hypothetical protein C0J52_26426 (*B. germanica*)	48.15	0.00012	694
4*	Putative Cytochrome P450 6a14 (*Blattella germanica*)	100% match to PSN34612.1 from *B. germanica* genome	39.40	0.00003	1142
5*	Cytochrome P450 6j1 (*Cryptotermes secundus*)	66% match to hypothetical protein C0J52_26426 (*B. germanica*)	34.22	0.00003	1095
6*	Cytochrome P450 6j1 (*Cryptotermes secundus*)	70% match to hypothetical protein C0J52_26426 (*B. germanica*)	33.82	0.00019	2042
7*	Cytochrome P450 6j1 (*Cryptotermes secundus*)	79% match to hypothetical protein C0J52_26426 (*B. germanica*)	33.54	0.00002	476
8*	Cytochrome P450 6j1 (*Cryptotermes secundus*)	100% match to hypothetical protein C0J52_26426 (*B. germanica*)	31.25	0.00054	531
9*	Cytochrome P450 6j1-like (*Cryptotermes secundus*)	81% match to hypothetical protein C0J52_26426 (*B. germanica*)	30.45	0.00037	1105
10*	Cytochrome P450 6k1-like (*Zootermopsis nevadensis*)	100% match to hypothetical protein C0J52_20551 (*B. germanica*)	17.44	0.00018	546
11	Putative Cytochrome P450 6a14 (*Blattella germanica*)	86% match to hypothetical protein C0J52_20551 ((*B. germanica*)	7.85	0.00018	2940
12	Cytochrome P450 6j1 (*Cryptotermes secundus*)	66% match to hypothetical protein C0J52_26426 (*B. germanica*)	6.83	0.00001	2807
13	Cytochrome p450 15F1 (*Reticulitermes flavipes*)	99% match to Methyl farnesoate epoxidase, partial (*B. germanica*)	6.81	0.00003	2003
14	Cytochrome P450 4C1; AltName: Full = CYPIVC1 (*Blaberus discoidalis*)	53% match to Cytochrome P450 4C1 (*B. germanica*)	6.33	0.00026	1974
15	Cytochrome P450 6j1 (*Cryptotermes secundus*)	85% match to hypothetical protein C0J52_26834 (*B. germanica*)	5.31	0.00000	2390
16	Cytochrome P450 4C1 (*Cryptotermes secundus*)	54% match to Cytochrome P450 4c21 (*B. germanica*)	5.13	0.00030	1687
17	1,5-anhydro-D-fructose reductase (*Cryptotermes secundus*) 75% match	63% match to 1,5-anhydro-D-fructose reductase (*B. germanica*)	4.91	0.00014	997
18	Per a allergen (*Periplaneta americana*)	63% match to Glutathione *S*-transferase (*B. germanica*)	4.11	0.00003	991
19	Cytochrome P450 6j1 (*Cryptotermes secundus*)	82% match to hypothetical protein C0J52_12805 (*B. germanica*)	3.83	0.00000	2083
20	Cytochrome P450 6j1 (*Cryptotermes secundus*)	48% match to Cytochrome P450 6j1 (*B. germanica*)	3.36	0.00003	1830
21	Peritrophic membrane protein 4, partial (*Holotrichia oblita*)	80% match to hypothetical protein C0J52_18875 (*B. germanica*)	2.64	0.00003	294
22	Venom carboxylesterase-6-like (*Zootermopsis nevadensis*)	99% match to hypothetical protein C0J52_16277 (*B. germanica*)	2.60	0.00082	3127
23	Cytochrome P450 6j1 (*Cryptotermes secundus*)	99% match to hypothetical protein C0J52_26834 (*B. germanica*)	2.30	0.00000	2568
24	Esterase FE4 (*Zootermopsis nevadensis*)	62% match to hypothetical protein C0J52_03840 (*B. germanica*)	1.77	0.00033	2177
25	Aldehyde dehydrogenase, partial (*Blattella germanica*) 100% match	Aldehyde dehydrogenase, partial (*B. germanica*) 100% match	1.70	0.00000	5007
26*	Chitinase-3-like protein 1 isoform X2 (Zootermopsis nevadensis)	73% match to hypothetical protein C0J52_01400 (*B. germanica*)	1.60	0.00060	2432
27	Chitin deacetylase 2 (*Nilaparvata lugens*)	100% match to hypothetical protein C0J52_26402 (*B. germanica*)	0.603	0.00038	2454
28	Bacterial aldo/keto reductase (Ruminococcus sp.)	No match	0.601	0.00058	2418
29	Cytochrome P450 4C1 (*Blattella germanica*) 100% match	Cytochrome P450 4C1 (*B. germanica*) 100% match	0.482	0.00082	2742
30*	Esterase FE4 (*Blattella germanica*) 99% match	Esterase FE4 (*B. germanica*) 99% match	0.405	0.00029	2208
31	Cytochrome P450 9e2 (*Zootermopsis nevadensis*)	78% similar to hypothetical protein C0J52_03714 (*B. germanica*)	0.381	0.00028	2335
32	Protist ERD2 (endoplasmic reticulum retention receptor) (*Symbiodinium necroappetens*)	No match	0.163	0.00044	4453
33*	Venom carboxylesterase-6 (*Blattella germanica*) 55% identity	Venom carboxylesterase-6 (*B. germanica*) 55% identity	0.153	0.00036	2132
34	Fungal membrane transporter (*Diplocarpon rosae*)	No match	0.142	0.00090	5851
35	GREGARINE piwi domain protein (*Gregarina niphandrodes*)	No match	0.136	0.00070	3424
36	Bacterial chitinase (*Legionella nagasakiensis*)	No match	0.123	0.00028	2674
37	PROTIST lysophospholipase II (*Nannochloropsis gaditana* CCMP526)	No match	0.113	0.00058	3625
38	GREGARINE chitinase (*Gregarina niphandrodes*)	No match	0.109	0.00026	1840
39	GREGARINE glutathione *S*-transferase (*Gregarina niphandrodes*)	No match	0.108	0.00022	2616
40	GREGARINE chitinase/lysozyme protein (*Gregarina niphandrodes*)	No match	0.107	0.00029	857
41	GREGARINE indolepyruvate decarboxylase (*Gregarina niphandrodes*)	No match	0.104	0.00022	2027
42	GREGARINE aldehyde dehydrogenase (*Gregarina niphandrodes*)	No match	0.101	0.00035	1772
43	Insect glutathione *S*-transferase 1-like (*Aricia agestis*)	No match	0.096	0.00051	4634
44	COCCIDIA ABC1 family protein (*Toxoplasma gondii* VEG)	No match	0.092	0.00028	4696
45	GREGARINE ATP-binding ABC transporter, partial (*Gregarina niphandrodes*)	No match	0.082	0.00078	2531
46*	GREGARINE superoxide dismutase (*Gregarina niphandrodes*)	No match	0.078	0.00044	830
47*	COCCIDIA phospholipase/carboxylesterase (*Toxoplasma gondii* GT1)	No match	0.072	0.00061	1338
48*	GREGARINE glutathione *S*-transferase (*Gregarina niphandrodes*)	No match	0.069	0.00026	2156
49*	BACTERIAL NAD-dependent formate dehydrogenase (*Granulicella* sp. S156)	No match	0.034	0.00086	1358
50*	Virus polyprotein 1 (Praha dicistro-like virus 2) 97% match	No match	0.013	0.00000	1897
51*	No match	No match	0.007	0.00001	1218
52*	Virus polyprotein 2 (Praha dicistro-like virus 2) 96% identity	No match	0.005	0.00000	2499
53*	Virus RNA-dependent RNA polymerase RdRp (Hubei permutotetra-like virus 8)	No match	0.001	0.00002	5487

The order shown is ranked from most highly upregulated (top) to most downregulated (bottom). All 53 contigs were tested in qRT-PCR validations against Illumina read count data, and a subset of 21 contigs indicated by asterisks* was used for post hoc regression comparisons between different strains (see Figure 6). Values shown are based on voom-limma analysis. See Supplementary Table 2 for primer sequences. ^1^nr database at ncbi.nlm.nih.gov/genbank/.

Expression of a subset of 21 genes (indicated by asterisks* in Table 1 and Supplementary Table 2) that showed significant differential expression in transcriptome analyses was also quantified in the lab-susceptible JWax-S and resistant Oviedo-R strains. These genes included 13 host cockroach genes (8 P450s, 2 carboxylesterases, 1 chitinase, 1 transposable element, and 1 hypothetical protein) and 8 genes from eukaryotic/viral microbiota (4 virus, 2 gregarine, 1 coccidia, and 1 unknown). Total RNA was extracted from 1 to 2 weeks old adult males in three replicate groups of 10 for the above two strains. RNA extractions were done using the two- step process described under “Transcriptome Sequencing.” cDNA synthesis was done from 500 ng total RNA using the iScript™ cDNA synthesis kit (Bio-Rad, Hercules, CA, United States). All qPCR procedures and expression analysis methods were similar to those mentioned above.

### Statistical Analyses

Probit analysis was used for LD and LC determinations using SAS software Version 9.2. Mortality data were corrected for control mortality (<10%) using Abbott’s transformation prior to conducting Probit analysis. Resistance ratios (RRs) were calculated by dividing LC or LD estimates for the selected lines by corresponding values for susceptible lines. Significance of RRs was determined according to the procedure outlined by Robertson and Preisler (1992). Mean-separation analyses of insecticide toxicity (vial diagnostic and no choice bait feeding bioassays) were done by ANOVA and paired t-tests (*P* < 0.05) after arcsine transformation of raw mortality data. For enzyme activity assays, specific activity values for the F6-selected and susceptible sub-populations were compared by non-parametric Mann–Whitney *U* tests (*P* < 0.05). Methods used for differential gene expression analysis of read-count data from transcriptome sequencing are explained in a separate section above. Regression analyses were done using JMP Pro 15 software (SAS Institute, Cary, NC, United States) to compare (i) qRT-PCR results to Illumina read counts for the F6-selected and control lines (*n* = 48 genes), and (ii) the F6-selected and control lines to each other and the Oviedo-R and JWax-S strains (*n* = 21 genes).

## Results

### Selection and Resistance Evolution

Six generations of selection of the parental AP strain with indoxacarb bait (Figure 1A) resulted in a selected strain having significant levels of resistance in both surface contact (23.8×; Figure 1B) and feeding bioassays (4.5×; Supplementary Table 2). The control strain left to inbreed without selection over the same timeframe acquired no resistance. The selection process led to heritable genetic changes with a realized heritability estimate (h2) of 0.28 (Supplementary Table 3). Biochemical assays on the F6-selected and control lines revealed >2× elevated esterase activity (*P* = 0.0004; Figure 1C) and ∼0.5× decreased P450 *O*-demethylation activity (*P* = 0.0004; Figure 1D).

### Metatranscriptome Sequencing and Assembly

From six total libraries representing three replicates each of the selected and control lines, 133 million paired-end sequence reads were obtained that contained >1.3 billion total nucleotide bases having an average read length of 98 base pairs (bp). The resulting overlapping sequences were assembled into 207,672 contiguous sequences, hereafter referred to as “contigs.” Sequence reads are deposited in the NCBI GEO archive under accession number GSE188950.

### Validation of *de novo* Transcriptome and Differentially Expressed Contigs

Contig length ranged from 201 to 30,113 bp with an average length of 1,777 bp and a N50 size of 4,805 bp; i.e., half of the assembled metatranscriptome is covered by contigs ≥ 4,805 bp. Next, a subset of 48 assembled contigs was chosen for validation analysis by qRT-PCR using the same RNA preparations as were used for Illumina sequencing (Table 1). A regression plot of log2 transformed Illumina transcriptome read count (X) vs. log2 transformed qRT-PCR Cq values (Y) revealed a highly significant correlation (*P* < 0.0001, *r*^2^ = 0.51) (Supplementary Figure 1). The latter result independently verifies the accuracy of the metatranscriptome results.

**FIGURE 2:**
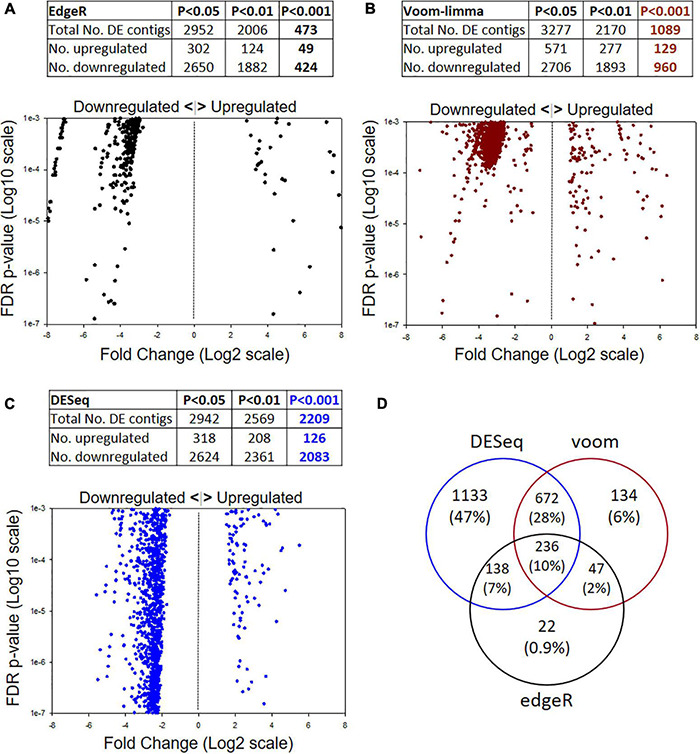
Illumina metatranscriptome analysis by three different models: **(A)** edgeR, **(B)** voom-limma and **(C)** DESeq. The top of each panel shows summary statistics for differentially expressed contigs and different false discovery rate (FDR) *p*-values. The bottom of each panel shows scatter plots of Log2 Fold Change (*x*) by *p*-value (*y*) for the FDR *P* < 0.001 datasets. **(D)** Venn diagram showing the numbers of passing contigs at the *P* < 0.001 level shared among different analysis models. The edgeR model was the most stringent at the *P* < 0.001 level.

DE analysis by three different models yielded differing numbers of passing contigs (Figure 2). Different FDR *p*-values were considered (*P* < 0.05, 0.01, 0.001) but the greatest emphasis is placed on the *P* < 0.001 level. The most stringent analysis model was edgeR with 473 passing contigs (Figure 2A), followed by voom-limma with 1,089 (Figure 2B) and DESeq with 2,209 (Figure 2C). Another interesting feature of the datasets for all three models was the higher ratio of downregulated:upregulated contigs in the selected strain; i.e., 424:49 for edgeR, 960:129 for voom-limma, and 2,083:126 for DESeq. Overall, there were 236 significant DE contigs shared among all three analysis models at the FDR *P* < 0.01 level and log2FC of ±1 (Figure 2D). Only contigs passing in two or more models at the FDR < 0.001 log2FC ±1 level were considered for further analysis as detailed below.

### GO Annotation and KAAS Pathway Analyses

BLAST2GO analyses for gene annotation revealed that 36.6% of contigs were annotated (76,053). For *P* < 0.001 passing contigs there were 779 GO terms for cellular location (CL; 99 upregulated, 680 downregulated), 1,514 for molecular function (MF; 209 up, 1,305 down) and 1,493 for biological process (BP; 265 up, 1,288 down) (Supplementary Figure 2A). CL terms potentially related to insecticide resistance include membrane, ribosome, mitochondria, microsome, endoplasmic reticulum and dendrite (Supplementary Figures 2B–D). Potentially relevant MF terms include electron carrier, hydrolase, monooxygenase, oxidoreductase, transferase and catalytic activity; and ATP, calcium, iron, heme, nucleotide and sugar binding. Lastly, relevant BP terms potentially linked to resistance include metabolic, transport, phosphorylation, glutathione conjugation and response to drug.

KAAS analysis was used to gain further insights into responsive pathways and gene networks (Supplementary Figure 3). Upregulated pathways potentially linked to resistance included drug metabolism by Cytochrome P450, fatty acid biosynthesis and pentose/glucuronate interconversion. Key downregulated pathways were related to microbial metabolism, viral infection, ribosome function/biogenesis, phagosomes, RNA degradation and epithelial cell signaling.

### Candidate Genes and Taxonomic Matches of Differentially Expressed Transcripts

**FIGURE 3:**
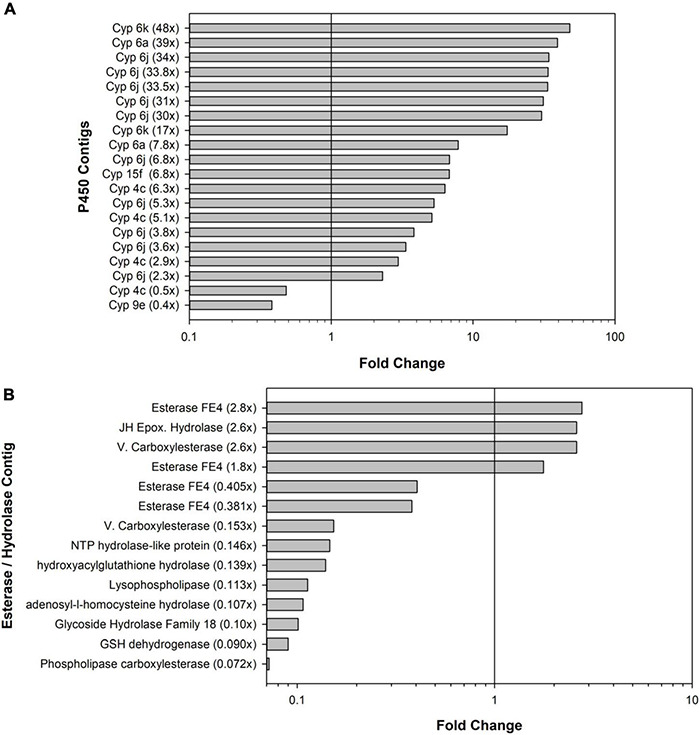
Bar graphs showing the numbers of significant differentially expressed contigs. **(A)** Cytochrome P450 (Cyp) contigs and **(B)** esterase and hydrolase contigs. Bars to the right of vertical black lines indicate upregulated contigs; bars to the left indicate downregulated contigs.

The blastx identities of many upregulated transcripts had logical links to resistance and detoxification. Upregulated cockroach genes included many gene families commonly associated with insecticide resistance including P450 oxidases and hydrolases (Figure 3) and glutathione-*S*-transferases (GSTs). Table 1 overviews the 21 contigs (shown by asterisks*) used for *post hoc* validations and gives a general overview of upregulated transcripts that mainly match the *B. germanica* genome, along with downregulated transcripts that originate mainly from microbial sources. The identities of sequences from either cockroach or microbial origins were re-verified by Genbank blastX searches performed in September 2021 (Table 1).

**FIGURE 4:**
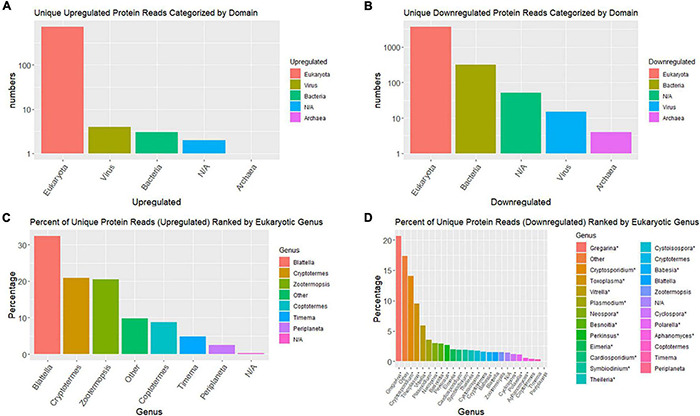
Bar graphs summarizing top taxonomic matches of top BlastX hits for differentially expressed contigs. **(A,B)** Are domain-level taxonomic matches and **(C,D)** are genus-level. **(A,C)** Are upregulated contigs and **(B,D)** are downregulated.

Taxonomic compositions of significant DE contigs (*P* < 0.001), based on blastx database queries, indicate the majority of upregulated contigs are from the cockroach genome; whereas, the majority of downregulated contigs come from viruses and eukaryotic microbes (Figure 4). Top upregulated taxonomic matches at the domain level were overwhelmingly eukaryotic whereas downregulated contigs had >10-fold more numbers of matches to bacteria, viruses and archaea than insects. At the genera level the top taxonomic matches for upregulated contigs were nearly all insects (*Blattella*, *Cryptotermes*, *Zootermopsis*, *Coptotermes*, *Timema*, and *Periplaneta*). Downregulated transcripts at the genus level were dominated by eukaryotic microbes that are apparently commensal, pathogenic and/or parasitic (*Gregarina*, *Cryptosporidium*, *Toxoplasma*, *Vitrella*, *Plasmodium*, *Neospora*, etc.).

### *Post hoc* Validations in an Independent Resistant Strain

**FIGURE 5:**
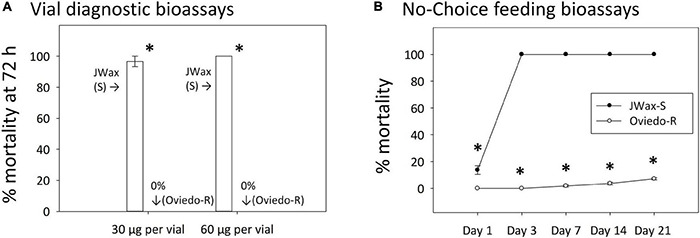
Indoxacarb bioassay results for the highly resistant Oviedo-R strain and the standard susceptible JWax-S strain. **(A)** Vial diagnostic concentration bioassays at two concentrations showing high-level mortality and the JWax-S strain and 0% mortality in the Oviedo-R strain. **(B)** No-choice feeding bioassays using commercial formulated indoxacarb bait showing rapid high-level mortality in the JWax-S strain and virtually no mortality in the Oviedo-R strain. *Asterisks indicate significant differences between strains at *P* < 0.0001.

An independent resistant strain, Oviedo-R, was collected from the field after indoxacarb bait control failures and assayed alongside the standard susceptible JWax-S strain. The Oviedo-R strain was highly resistant with 0% mortality in vial bioassays on diagnostic concentrations of indoxacarb (Figure 5A). These diagnostic concentrations were approximately two and fourfold higher than the indoxacarb LC50 for the Control (F6) strain shown in Figure 1B. The JWax-S strain was highly susceptible with 100% mortality in the same assays. When tested in no-choice feeding bioassays with commercially-formulated indoxacarb bait, the Oviedo-R strain displayed exceptionally high resistance (ANOVA df = 1,48, *F* = 130.50, *P* < 0.001) (Figure 5B). The Oviedo-R strain entirely consumed 0.5 g indoxacarb bait per assay replicate by Days 7 and 14, at which time the bait was replenished. These results show high levels of physiological resistance to indoxacarb in the Oviedo-R strain with no involvement of a behavioral “aversion” component.

**FIGURE 6:**
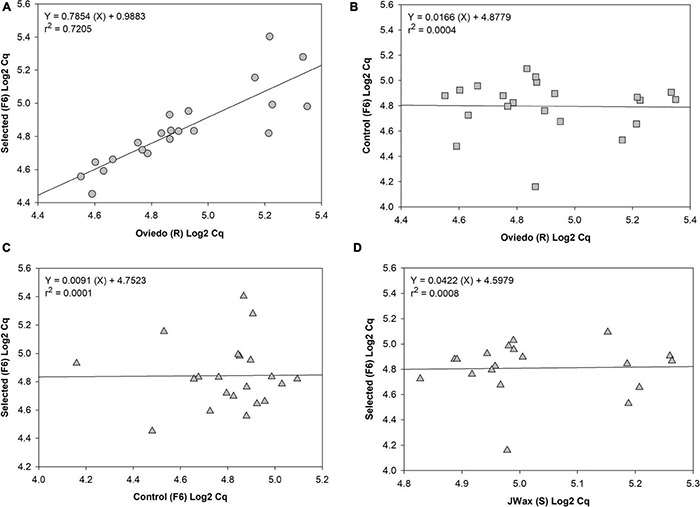
Regression analyses of qRT-PCR relative expression data for 21 significant differentially expressed contigs. Results show **(A)** well-correlated gene expression between the selected-F6 and highly resistant Oviedo-R strains, but no correlation when comparing **(B)** Oviedo vs. Control-F6, **(C)** Control-F6 vs. Selected-F6, or **(D)** JWax-S vs. Selected F6. All *C*q data were Log2 transformed before performing regressions.

Finally, to test for common patterns of gene expression across strains, qRT-PCR analyses were performed on a subset of 21 significant up- and down-regulated contigs identified from the transcriptome analysis presented above (Table 1 and Supplementary Table 2). The four strains included in this analysis were Oviedo-R, JWax-S, indoxacarb Selected-F6 and Control-F6. From a series of regression analyses, there was significantly correlated transcript abundance between the Selected-F6 and Oviedo-R strains (Figure 6A), but no correlation when comparing Oviedo-R vs. Control-F6 (Figure 6B), Control-F6 vs. Selected-F6 (Figure 6C), and JWax-S vs. Selected F6 (Figure 6D). These results suggest common processes associated with indoxacarb resistance evolution in lab and field-selected cockroach populations.

## Discussion

### Overview

This study reveals new insights into cockroach insecticide resistance evolution from multiple physiological perspectives. Through a combination of approaches including selection for resistance via feeding on insecticidal bait, coupled with different bioassay formats and metatranscriptome sequencing, we were able to observe that (1) the host cockroach mainly upregulates a range of detoxification resistance mechanisms, and (2) at the same time decreases its internal virus, parasite and/or pathogen levels. Further investigation of candidate gene expression in the highly resistant Oviedo-R strain suggests common phenomena that underlie resistance evolution. Taken together, these findings support the idea that high-level resistance evolution results from a dual process whereby the host tolerates the selecting insecticide through a number of potential mechanisms, and in parallel, host fitness is further increased as the body is cleared of parasites and pathogens. At present it remains unclear if the microbial impacts result from (i) direct effects of indoxacarb, (ii) indirect effects of antimicrobial preservatives included in the selecting bait matrix, or (iii) selection for general stress response mechanisms that confer both xenobiotic resistance and microbial immunity. With respect to the first two possibilities, it is unclear if these processes are associated generally with all insecticides, or specifically with ingested indoxacarb.

Transcriptome sequencing revealed dozens of candidate upregulated genes from the host cockroach potentially involved in resistance, including detoxification enzymes (discussed below), transport mechanisms, host gut penetration barriers, and others. An unanticipated result was the downregulation thereby indicating disappearance of commensal, pathogenic and/or parasitic microbe transcript contigs after insecticide selection (also discussed below). Findings in both categories are corroborated by the GO and KAAS analyses, which provide additional independent confirmation for the sequence composition results relating to detoxification and parasite/pathogen disappearance. The qPCR correlation analyses between resistant and susceptible strains lends further strength to the above findings. Specifically, the regression analyses showed similar trends between independently-selected resistant strains for upregulation and downregulation of host detox mechanisms, and disappearance of a wide range of microbes. However, there appears to be wider variation with respect to disappearance of microbial contigs (right side of Figure 6A). This finding is logical given that different environments have different microbiomes associated with them that can, in turn, shape internal microbiomes of higher organisms living within them (Renelies-Hamilton et al., 2021; Tinker and Ottesen, 2021).

### Detoxification and Metabolism Mechanisms

In terms of detoxification and metabolism, indoxacarb is a unique pro-insecticide that requires hydrolytic activation to its decarbomethoxyllated “DCJW” metabolite to become an active insecticide (Wing et al., 2000; Alves et al., 2008). A prior study investigating indoxacarb metabolism pathways in resistant and susceptible *B. germanica* strains revealed that hydrolysis and P450-oxidation were important to activation and resistance-associated detoxification, respectively (Gondhalekar et al., 2016). The current study reveals candidate genes involved in both oxidative and hydrolytic metabolism of indoxacarb; specifically, 20 differentially expressed (DE) P450 contigs and 14 DE esterase/hydrolase contigs. In theory, resistance could result from both upregulation of P450 oxidative enzymes and downregulation of hydrolases, both of which were detected in the present study.

With respect to detoxification genes and the *B. germanica* genome, a targeted analysis revealed 158 P450 genes total, which represents some gene expansions in relation to the available genomes of close insect relatives (Harrison et al., 2018a,b). The most expanded P450 families were Cyp4 (*n* = 59), followed by a subset of Cyp6 (*n* = 8). The present study identified resistance-associated changes of expression of 12.6% of the *B. germanica* “cypome,” with the majority (14) being Cyp6 members and fewer being Cyp4s (4). It is also noteworthy that a transposable element potentially associated with Cyp4 expansion (Harrison et al., 2018b) had higher resistance-associated expression in both the indoxacarb-selected line and the field-selected Oviedo-R strain. Expansions previously identified for other detoxification genes in the *B. germanica* genome included 62 E4 esterases and a subgroup of 23 GST genes (Harrison et al., 2018b). The present study identified 14 DE esterases (4 up- and 10 down-regulated) and 11 GSTs (6 up- and 5 down-regulated). Significant changes in both P450 and esterase activity were noted with selection (Figure 1) but not GST activity (Gondhalekar, 2011).

Numerous DE P450 contigs identified here were close homologs to Cyp6J1 and 6K1 of *B. germanica* (Wen and Scott, 2001), but none were identical matches. Some of these Cyp6 homologs had 20–50× upregulation with indoxacarb selection. Based on these results it is likely that there is Cyp6 involvement in the detoxification pathway for indoxacarb (Gondhalekar et al., 2016) and more Cyp6 diversity in the *B. germanica* genome than initially suggested (Wen and Scott, 2001; Harrison et al., 2018b). A previous study found Cyp4G19 over expression in association with fipronil and imidacloprid resistance in *B. germanica*, but not indoxacarb resistance, which agrees with results of the present study finding no change of expression for Cyp4G P450s. Cyp4G19 was also linked to pyrethroid resistance, cuticular hydrocarbon production and cuticular penetration-based resistance (Pridgeon et al., 2003; Guo et al., 2010; Pei et al., 2019; Chen et al., 2020; Hu et al., 2021), which are seemingly unrelated to the ingestion and gut uptake that occurred during the six generations of indoxacarb selection that were done in the present study.

The current study also identified DE homologs of Cyp9e2 and Cyp4C21 with possible roles in indoxacarb biotransformation. Both Cyp9e2 and Cyp4C21 were previously identified from *B. germanica* along with apparent homologous pseudogenes that may confound gene identification (Wen et al., 2001). Another upregulated P450 in the present study was Cyp15F1, which catalyzes the last step in juvenile hormone biosynthesis (Maestro et al., 2010; Tarver et al., 2012; Zhou et al., 2014). The Cyp15F1 homolog identified in the present study had a strong match to its corresponding genomic sequence reported in the *B. germanica* genome (Harrison et al., 2018a,b). It is not clear if Cyp15F1 participates in indoxacarb detoxification or another physiological process related to gut tissue remodeling after indoxacarb exposure, or in response to clearance of gut microbes (e.g., Sen et al., 2015). A previous study identified the P450 protein “P450MA” from a multi-resistant *B. germanica* strain (Scharf et al., 1998). P450MA was overexpressed after organophosphate insecticide selection and also was found to be over-expressed in organophosphate resistant *B. germanica* strains from different global origins (Scharf et al., 1997b,^1999^). While the molecular identity of P450MA remains unknown, it may be among the P450s identified in the present study, of which Cyp6 members were the most abundant.

Lastly, the identification of decreased P450 *O*-demethylation activity in the F6-selected strain does not appear to be an erroneous or trivial result. This is because there is good agreement between reduced *O*-demethylation activity in our F6-selected indoxacarb-resistant strain and transcriptome results showing downregulation of several P450 contigs in the same genetic lineage. This latter finding also suggests that increased *O*-demethylation activity may not be a useful biomarker for indoxacarb resistance.

With regard to hydrolytic activity, it is considered more important for indoxacarb activation in *B. germanica* than detoxification (Gondhalekar et al., 2016). Esterases and associated hydrolases are overall not as well studied as the P450s discussed above, but several DE hydrolase and esterase homologs were identified through the current study. Several of these DE esterase contigs had significant matches to FE4 esterases from other insects with well-established roles in insecticide metabolism (Field and Foster, 2002; Srigiriraju et al., 2009). Esterase activity toward model substrates was also elevated in the F6-selected strain, which is consistent with the several upregulated esterase/hydrolase contigs that were identified through transcriptome sequencing. It is also possible that, despite being important for indoxacarb activation, increased hydrolytic activation could still enable greater detoxification by P450 and other detox enzymes and subsequent clearance from the body (Gondhalekar et al., 2016) by Glutathione-*S*-transferases, some of which were also upregulated.

### Microbial Disappearance

Invertebrates were the first hosts of apicomplexan parasites like *Plasmodium* and *Toxoplasma* which later switched to vertebrate hosts during their evolution (Kopecná et al., 2006). Biotic associations of cockroaches with microbes have been known for over 100 years and include bacteria, archaea, viruses, protists, fungi, gregarines, and nematodes (Roth and Willis, 1960; Kakumanu et al., 2018). In the present study, transcript contigs from representatives of all of these groups were greatly reduced with indoxacarb selection. Omics approaches similar to those used here have previously identified gregarines and other eukaryotic microbes in insects and thus it should not be surprising that similar eukaryotic microbe transcripts were identified in the present study through the use of poly-A RNA tagging (McCarthy et al., 2011; Scharf, 2015; Scharf et al., 2017). Many of the microbial genera that were reduced by the selection process are potential pathogens to humans and companion animals (e.g., *Cryptosporidium*, *Toxoplasma*, *Vitrella*, *Plasmodium*, *Neospora*, etc.) and thus our findings on their disappearance may be highlighting a previously unknown/unacknowledged benefit of cockroach baits.

Gregarine protist contigs were the dominant downregulated contigs in the F6-indoxacarb selected line. One common parasitic gregarine, *Gregarine blattarum*, can infect multiple cockroach species including *B. germanica*, as well as other invertebrates (Yahaya et al., 2017). Gregarine infection has been shown to cause pathological effects in *B. germanica* as well as increase susceptibility to the fungal pathogen *Metarhizium anisopliae* and the growth regulator insecticide triflumuron (Lopes and Alves, 2005), which compels us to ask the question: *is insecticide susceptibility caused in part by microbial pathogens or parasite stress*? Cockroaches in culture are particularly susceptible to gregarine infections and such infections can be reduced by antimicrobial drugs (Clopton and Smith, 2002; Smith and Clopton, 2003). However, it does not appear that many of the sampled microbial groups are exclusively associated with laboratory rearing (Kakumanu et al., 2018). Gregarines are further known to accelerate larval development in cat fleas, *Ctenocephalides felis* (Alarcón et al., 2017) and have recently been identified in cucumber beetles, *Acalymma vittatum*, with no apparent impacts on fitness (Coco et al., 2020). To our knowledge no prior reports are available describing the effects of insecticides on gregarines, but a prior report does document impacts on related termite gut protists by the nicotinoid insecticide imidacloprid (Sen et al., 2015). While it is not clear if the disappearance of these internal microbes enhances host fitness to enable a rapid buildup of xenobiotic resistance, or *vice-versa* (i.e., if resistance happens first), our findings provide important new insights into the potential stepwise basis of resistance evolution and the complex physiological interactions involved.

## Conclusion

This study provides new insights into cockroach insecticide resistance evolution from multiple physiological perspectives ranging from xenobiotic metabolism and excretion to pathogen and parasite resistance. The identities of dozens of candidate bioactivation and detoxification enzymes were revealed, namely cytochrome P450s and esterases/hydrolases, which agrees strongly with outcomes of preceding indoxacarb metabolomics work in *B. germanica* (Gondhalekar et al., 2016). Many of the genes studied here also had strong matches to the *B. germanica* genome (Harrison et al., 2018a,b) and thus our findings provide important annotations that have been lacking in many cases. Because we used a selection-based approach with temporally parallel non-selected controls that originated from the same genetic stock, and compared strains from both common and distinct genetic origins in a stepwise fashion, the insights provided have limited caveats. Thus, the correlated expression for a subset of candidate genes between independently-selected resistant strains suggests there are common, predictable patterns to resistance evolution across populations.

An unanticipated outcome was that numerous microbial transcripts were reduced with insecticide selection. In terms of the causative agents behind microbial disappearance, three possibilities exist: (i) direct antimicrobial effects by indoxacarb, (ii) indirect effects of antimicrobial preservatives in the bait matrix, or (iii) co-selection for dual detoxification and immune pathways. While the potentially direct effects of the selecting insecticide or bait matrix preservatives on eukaryotic microbes are logical, the causative factors underlying virus and bacterial declines are less clear and should be further investigated. Findings also revealed a potential added benefit of cockroach baits for curing cockroaches of potentially deleterious pathogens and associated allergens that can affect both humans and companion animals. *A priori* goals of this study did not include identification of effects on cockroach parasites and pathogens, and thus our experimental design was not optimized for gaining insights into host-microbial interactions. Future work thus needs to examine for similarities in genes, microbes and processes revealed here, with the ultimate goal of reducing impacts of insecticide resistance and concurrently creating healthier indoor urban environments.

## Data Availability Statement

The datasets presented in this study can be found in provided Supplementary Material, as well as in online repositories. The names of the repository/repositories and accession number(s) can be found below: NCBI GEO, accession no: GSE188950.

## Author Contributions

MS designed the research, performed data analysis, and drafted the initial manuscript. ZW performed taxonomic analyses. KR and MF conducted qPCR analyses. JT and KB performed bioinformatics analyses and statistics. AG designed and conducted the research, performed data analyses, and edited the manuscript. All authors contributed to the article and approved the submitted version.

## Conflict of Interest

This study received gift funding from DuPont Inc. The funder was not involved in the study design, collection, analysis, interpretation of data, the writing of this article or the decision to submit it for publication. The authors declare that the research was conducted in the absence of any commercial or financial relationships that could be construed as a potential conflict of interest.

## Publisher’s Note

All claims expressed in this article are solely those of the authors and do not necessarily represent those of their affiliated organizations, or those of the publisher, the editors and the reviewers. Any product that may be evaluated in this article, or claim that may be made by its manufacturer, is not guaranteed or endorsed by the publisher.

## Footnotes

1
http://bioinfogp.cnb.csic.es/tools/venny/index.html

2
https://bioinfo.ut.ee/primer3-0.4.0/

## Supplementary Material

Supplementary Table 1 Supplementary Tables & Figures.

Supplementary Table 2 Upregulated contig summary statistics.

Supplementary Table 3 Downregulated contig summary statistics.

Supplementary Data Sheet 1 FASTA sequences of upregulated contigs.

Supplementary Data Sheet 2 FASTA sequences of downregulated contigs.
