# Supplementary material for: Transcriptome Responses to Defined Insecticide Selection Pressures in the German Cockroach (Blattella germanica L.)
Source: Front Physiol. 2022 Feb 4;12:816675. doi: 10.3389/fphys.2021.816675 (PMC8856671; doi:10.3389/fphys.2021.816675)
Supplement: Supplementary Table 1 — Supplementary Tables & Figures. [file Table_1.DOCX]

**SUPPLEMENTARY INFORMATION**

**Table S1.** Summary of indoxacarb selection experiments with large nymphs (4^th^–5^th^ instar) of three individual lines isolated from the Parental (F0) AP strain. Average numbers of nymphs subjected to indoxacarb selection per generation was >600 (represents the sum of three selected lines or replicates).

| **Generation** | **Oral Dose**  **(µg/insect)** | **% Survival** | | |  |
| --- | --- | --- | --- | --- | --- |
|  |  | **Line 1** | **Line 2** | **Line 3** |  |
| Parental F0 | 1.0 | 26.12 | 25.70 | 19.46 |  |
| F1 selected | 1.0 | 37.81 | 43.60 | 42.31 |  |
| F2 selected | 1.25 | 31.46 | 31.31 | 32.54 |  |
| F3 selected | 1.25 | 37.72 | 33.54 | 39.44 |  |
| F4 selected | 2.5 | 25.92 | 35.85 | 47.50 |  |
| F5 selected | 3.5 | 30.71 | 39.61 | 39.61 |  |
| Average % survival from parental to F5 generation  (± SE) | | **27.85**  **(1.64)** | **28.98**  **(2.38)** | **32.23**  **(2.98)** | |

**Table S2**. Quantitative Real-Time PCR primers. Primer numbers with gray shading are for downregulated contigs; no shading indicates upregulated contigs. All primers were used for the first qPCR validation experiment and those with asterisks (*) indicate the subset that was used for the second qPCR experiment comparing independent strains.

| **Prim-er No.** | **Fasta Sequence ID** | **Primer set name** | **Forward primer sequence** | **Reverse primer site**  **cDNA sequence >>>** | **Reverse primer sequence (reverse complement of column to left)** |
| --- | --- | --- | --- | --- | --- |
| 1* | >001174-001179_All_comp203_c0_seq1 | U-CHI-1.6 | TAAGGTCCAGCAGTTCCAGC | GGAGCACCTTCACTCAACCA | TGGTTGAGTGAAGGTGCTCC |
| 2 | >001174-001179_All_comp713_c0_seq1 | U-ALD-DH-1.7 | AGCAGCAAGGCCATAGTCTG | AGCAAACACAGTGGGTTGGA | TCCAACCCACTGTGTTTGCT |
| 3 | >001174-001179_All_comp1322_c0_seq1 | U-JHE-1.77 | AATGCTCTTGTCGACTGCCT | CTACACCTCTGGAACTGGCG | CGCCAGTTCCAGAGGTGTAG |
| 4 | >001174-001179_All_comp293_c1_seq3 | U-CP-6K1-2.3 | CGCTCCGGATCGTACTTCTC | AGTGCACTTTCGGTCAAGGA | TCCTTGACCGAAAGTGCACT |
| 5 | >001174-001179_All_comp648_c0_seq1 | U-EST-2.6 | ACAGCCCAGAAGAGCTCCTA | GAAGTGGACGCTAGCTCCTC | GAGGAGCTAGCGTCCACTTC |
| 6 | >001174-001179_All_comp58_c0_seq2 | U-PERI-2.6 | CTTTGGGCAGTTGTCTTGGC | GCATCACCGGAATGAGGGAA | TTCCCTCATTCCGGTGATGC |
| 7 | >001174-001179_All_comp691_c2_seq1 | U-CP-6K1-3.3 | CTGGGTCGAACTTTGCTGGA | CTAGGCAGCTGGAAGTCGTT | AACGACTTCCAGCTGCCTAG |
| 8 | >001174-001179_All_comp374_c2_seq7 | U-CP-3.8 | GCGCGATTCACAACTGACAT | CTAGGCTGCAGGTCGTAAGG | CCTTACGACCTGCAGCCTAG |
| 9 | >001174-001179_All_comp1221_c0_seq1 | U-GST-D1 | GCATAGTATGGCATGGCTTTCC | AATTCAGCTCCACTCCCAACA | TGTTGGGAGTGGAGCTGAATT |
| 10 | >001174-001179_All_comp880_c0_seq2 | U-Ald-Keto-Rd | CAACCATCAAACCCGCAGTG | TAAGGAAGATACGCCCGCAC | GTGCGGGCGTATCTTCCTTA |
| 11 | >001174-001179_All_comp8441_c0_seq1 | U-CP-5.1 | ATGGTGGCTGGGAGGAATTC | TCCTTGGCTTGGAAAGGGTC | GACCCTTTCCAAGCCAAGGA |
| 12 | >001174-001179_All_comp834_c0_seq1 | U-CP-5.2 | TGAGAAGCACAGTCTGGGAC | TCATCGCCCAAGAATGACGA | TCGTCATTCTTGGGCGATGA |
| 13 | >001174-001179_All_comp15145_c0_seq1 | U-CP-6.3 | GTTCCTAACCACGGCTCCAA | GCTCGCGTGTTGTTCTTCAG | CTGAAGAACAACACGCGAGC |
| 14 | >001174-001179_All_comp1882_c2_seq3 | U-CP-6.8 | GACCTCGCGAAGTTCTGGAA | GCAACGTCGCTTTACTCTGAG | CTCAGAGTAAAGCGACGTTGC |
| 15 | >001174-001179_All_comp441_c0_seq1 | U-CP-6K1-6.9 | AAGAGCAAGTGGGAACGGAG | ACCGAACGGCATGTAAGTGT | ACACTTACATGCCGTTCGGT |
| 16 | >001174-001179_All_comp353_c1_seq1 | U-CP-7.8 | CTTTGGTGAAGGGCCTAGGT | AACACCCGTGCCAATGGAAT | ATTCCATTGGCACGGGTGTT |
| 17* | >001174-001179_All_comp7424_c0_seq3 | U-CP-17 | AGGGTCATGATGGAGGGACA | TCTGAGCGGGTGAGTGTTTC | GAAACACTCACCCGCTCAGA |
| 18* | >001174-001179_All_comp17842_c0_seq4 | U-CP-30 | TGCACACCCTAGGACCATCT | TTGGGTCGTGATGAAGTCCC | GGGACTTCATCACGACCCAA |
| 19* | >001174-001179_All_comp25393_c0_seq1 | U-CP-6A1-31 | CACCCACATTCACGTCAGGA | AACGGACGTCATTGCTTCTTG | CAAGAAGCAATGACGTCCGTT |
| 20* | >001174-001179_All_comp13050_c0_seq2 | U-CP-33.5 | TGAGGATTACGCAGCTCAGG | ACGCCTGAGTTCCGAAATCA | TGATTTCGGAACTCAGGCGT |
| 21* | >001174-001179_All_comp834_c0_seq2 | U-CP-33.8 | TGAGAAGCACAGTCTGGGAC | TCATCGCCCAAGAATGACGAT | ATCGTCATTCTTGGGCGATGA |
| 22* | >001174-001179_All_comp441_c0_seq2 | U-CP-6K1-34 | GGGTCCTTGAGTGTGTTGCT | GGTAGCCGTCCAGTTCCATC | GATGGAACTGGACGGCTACC |
| 23* | >001174-001179_All_comp7424_c0_seq1 | U-CP-39 | TCCATTTCGACCCGGAAAGA | GGGTTGGTTTGTCAAAGCGG | CCGCTTTGACAAACCAACCC |
| 24* | >001174-001179_All_comp2359_c0_seq5 | U-CP-48 | CCAGCCTACTCATACATGCCA | AGGACACACCGATTCCACTTC | GAAGTGGAATCGGTGTGTCCT |
| 25* | >001174-001179_All_comp790_c1_seq1 | U-NA-67 | AGCCCTTCCTAATGTCCCAATG | ACCAACGTCTATGCTCGAGG | CCTCGAGCATAGACGTTGGT |
| 26* | >001174-001179_All_comp1069_c0_seq3 | U_NA_84 | GTGCGGGGTATTCGTAACCT | TCAGCCGCTATGAACAGGTG | CACCTGTTCATAGCGGCTGA |
| 27 | >001174-001179_All_comp1071_c0_seq1 | D-Chi-Dace-1 | GTCCGTGCTCCTTATCTCCG | ATGTACTTCCGCATGCCACA | TGTGGCATGCGGAAGTACAT |
| 28 | >001174-001179_All_comp608_c0_seq1 | D-Al-KETO-RD | CCAACCCAGCCTACTTACCG | AAGCACTGCATGGATGACG | CGTCATCCATGCAGTGCTT |
| 29 | >001174-001179_All_comp4892_c0_seq1 | D-CP-2.1 | TTTCTCCGACCTCCTCCGAA | AGATGTGAACGACGCTTCCA | TGGAAGCGTCGTTCACATCT |
| 30* | >001174-001179_All_comp8500_c0_seq1 | D-EST-2.46 | ACCATCACTGCTGCTTGTTCA | CATATGCCCTTCTTGCTGAGC | GCTCAGCAAGAAGGGCATATG |
| 31 | >001174-001179_All_comp9442_c0_seq1 | D-CP-2.62 | CACGACTTCTTCCCTGGCTT | GCTGCCACATCTACATCGGT | ACCGATGTAGATGTGGCAGC |
| 32 | >001174-001179_All_comp11451_c0_seq1 | D-ER-RET | TCCATATTCGCTCGCTCCAC | TTGTCACTGAAGAGGCGACC | GGTCGCCTCTTCAGTGACAA |
| 33* | >001174-001179_All_comp35594_c0_seq1 | D-Ven-EST | GCTGTCCTACAACCACAAGC | ACAGACCAACAATTGAGGCAGT | ACTGCCTCAATTGTTGGTCTGT |
| 34 | >001174-001179_All_comp19442_c0_seq1 | D-Mem-Tr | AACAAGGTGGCATAGCACGA | AAGAGTGTGCACGCTGTACA | TGTACAGCGTGCACACTCTT |
| 35 | >001174-001179_All_comp24090_c0_seq1 | D-Argonaute-2 | GAAGGTTGATGCCGTCGTTG | AAGTCGATTTGGCCTCCCTG | CAGGGAGGCCAAATCGACTT |
| 36 | >001174-001179_All_comp7274_c0_seq1 | D-Class i_Chitinase | GACCTTGGAGACTGGACTGC | ACTCCCAGACAAAGCGGAAC | GTTCCGCTTTGTCTGGGAGT |
| 37 | >001174-001179_All_comp25274_c0_seq1 | D-Acyl.Prot.-EST | CCTACGTTGACTACCGCCTC | GACTTGATTGGGAGGCGGAA | TTCCGCCTCCCAATCAAGTC |
| 38 | >001174-001179_All_comp9485_c0_seq1 | D-Endo-chitinase | CTTGTGACGAACCGGGAAGA | AACGATGTTTCGAGCGGTCT | AGACCGCTCGAAACATCGTT |
| 39 | >001174-001179_All_comp7133_c0_seq1 | D-GST-Sigma | CCGCCATAGTCAAGCTTTGC | CCGACAAGGAGGAAAGGCAT | ATGCCTTTCCTCCTTGTCGG |
| 40 | >001174-001179_All_comp1147_c0_seq1 | D-Chitinase | TCAGGGAGCTTGTGATGCTG | GTCCACAACAACCACTTCGC | GCGAAGTGGTTGTTGTGGAC |
| 41 | >001174-001179_All_comp6368_c0_seq1 | D-INP-DC | TACATTCGCTGATGACCCGG | GCGTAGAATTTGCGAGCTGG | CCAGCTCGCAAATTCTACGC |
| 42 | >001174-001179_All_comp5121_c0_seq1 | D-ALDH-9.85 | ATTGTCCCAGTGCCGGATTT | CCGCTGCGAAAGTGATGAAC | GTTCATCACTTTCGCAGCGG |
| 43 | >001174-001179_All_comp11089_c0_seq1 | D-GST-10.46 | GCTAGGAAAGGTTCACGGCT | TCTGAGCAAGCCTTGACGAG | CTCGTCAAGGCTTGCTCAGA |
| 44 | >001174-001179_All_comp27495_c0_seq1 | D-ABC1-10.83 | CAGAATGTCGCCCTCCTCTC | GGCAAGTCGAGTCACTGTCA | TGACAGTGACTCGACTTGCC |
| 45 | >001174-001179_All_comp20826_c0_seq1 | D-ABC-12.2 | AAGCCTGACCAGAATCCAGC | GGATTCGTGGGCTACAGAGG | CCTCTGTAGCCCACGAATCC |
| 46* | >001174-001179_All_comp5448_c0_seq1 | D-SOD | ATCCTTGAGACCCATGACGC | TGGTGGAAGCTCGTCAACTG | CAGTTGACGAGCTTCCACCA |
| 47* | >001174-001179_All_comp30958_c0_seq1 | D-PHL-EST | AGAAGGTCTTGAATGCCCGG | TGCACACATGGCTTGCTCTA | TAGAGCAAGCCATGTGTGCA |
| 48* | >001174-001179_All_comp4155_c0_seq1 | D-GST-14.5 | CACTCTGCTCAGCGATGACA | AGCGTGGCACATGAATCTGA | TCAGATTCATGTGCCACGCT |
| 49* | >001174-001179_All_comp15414_c0_seq1 | D-Formate-DH | CTCCAGCATACCCAGCTAGC | TTGATTGAGGAGCTTGGCGT | ACGCCAAGCTCCTCAATCAA |
| 50* | >001174-001179_All_comp54121_c0_seq1 | D-NA-75 | CGAATGCGCCAAGAACGAAA | GGTTTCTTCTTGTTGCGCGT | ACGCGCAACAAGAAGAAACC |
| 51* | >001174-001179_All_comp29484_c0_seq1 | D-NA-144 | TGAAACGGGCACATAGTCGG | GTTCCTGCCGCCAGAGATTA | TAATCTCTGGCGGCAGGAAC |
| 52* | >001174-001179_All_comp39252_c0_seq1 | D-Struc-Prot | TCCAACAGCGTGTCAGAGTC | TGCACCAGTGTATGACCAGG | CCTGGTCATACACTGGTGCA |
| 53* | >001174-001179_All_comp9862_c0_seq1 | D-Coat_Prot | ACATACTGGGCACAACTGGG | CCAAGGAAACATTGCGTGCA | TGCACGCAATGTTTCCTTGG |

**Table S3.** Indoxacarb oral lethal dose (LD) estimates for the AP selected and susceptible strains at 72 h.

| **Strain** | **n** | **Chi-square**  **(df) ^a.^** | **Slope**  **(± SE)** | **LD_50_**  **(95 % FL) ^b.^** | **Resistance Ratio** |
| --- | --- | --- | --- | --- | --- |
| Selected (F6) | 630 | 2.44  (4) | 2.72  (0.19) | 0.72  (0.64 – 0.81) | 4.50*  (3.34 – 6.06) |
| Control (F6) | 210 | 4.04  (4) | 3.65  (0.53) | 0.16  (0.13 – 0.20) | 1.00 |
| Parental (F0) | 624 | 4.86  (4) | 2.75  (0.23) | 0.16  (0.14 – 0.18) | -.-- |
| n = total number of adult males used in bioassays.  ^a.^ Chi-square values and corresponding degrees of freedom.  ^b.^ Oral lethal dose (LD_50_) values at 72 h with 95% fiducial limits. Values are expressed in μg indoxacarb per insect. Average weights of all strains were similar; ca. 52 mg per adult male.  ^c.^ Resistance ratios at LD50 determined based on LD50 Selected or Control strains ÷ LD50 Parental strain. * Indicates significance based on non-overlap of LD fiducial limits. | | | | | |
|  | | | | | |

**Table S4.** Realized heritability (h^2^) of indoxacarb resistance in the Selected strain during the selection process. Results are determined from feeding bioassays detailed in Table S2).

| **Selection regime ^a.^** | **R ^b.^** | **1/R ^c.^** | **i ^d.^** | **σp ^e.^** | **S ^f.^** | **h^2 g.^** |
| --- | --- | --- | --- | --- | --- | --- |
| F0 to F5 | 0.11 | 9.00 | 1.08 | 0.36 | 0.39 | 0.28 |
| ^a.^ For heritability calculations, oral lethal dose estimates and associated parameters of the F1-susceptible and F6-selected lines were used.  ^b.^ Response to selection, R = [(log_10_ of final LD_50_ - log_10_ of initial LD_50_) ÷ number of generations selected].  ^c.^ Number of generations necessary for 10-fold increase in LD_50_ value or resistance level.  ^d.^ Intensity of selection, i = table value from Appendix B (Falconer 1989). This value is based on the average percentage of insects surviving selection regime over n number of generations (6 generations for present study). See Table 1 for average survival details.  ^e.^ Phenotypic standard deviation, σp = [1÷ (0.5 x (initial slope + final slope)].  ^f.^ Selection differential, S = i σp.  ^g.^ Realized heritability, h^2^ = R/S (Tabashnik 1992, Falconer 1989). | | | | | | |

**Supplementary Fig S1**. Validation of Illumina transcriptome results (X axis) by linear regression analysis against qRT-PCR analysis results (Y axis). A subset of 48 up- and down-regulated contigs were chosen for this analysis. Results shown in the plot are the log2 transformed read count values from illumina sequencing (X) and log2 transformed Cq values from qRT-PCR analyses (Y). Regression analysis was performed in JMP Pro with the results shown in the upper right corner of the resulting graph.

**Supplementary Fig S2**. Gene Ontology (GO) analysis results as determined using the BLAST2GO analysis tool (Conesa et al. 2005). (A) An overall summary of up- and down-regulated terms shared among passing differentially expressed contigs (P<.0001). (B-D) Summary of cellular location, molecular function and biological process terms among up- and down-regulated contigs.

**Supplementary Fig S3**. Summary of pathway analysis results obtained for significant differentially expressed contigs using the KAAS analysis tool (Moriya et al. 2007).
